# Rotation osteotomy of the distal femur influences coronal femoral alignment and the ischiofemoral space

**DOI:** 10.1007/s00402-020-03704-z

**Published:** 2020-12-23

**Authors:** Christian Konrads, Marc-Daniel Ahrend, Myriam Ruth Beyer, Ulrich Stöckle, Sufian S. Ahmad

**Affiliations:** 1 Department for Trauma and Reconstructive Surgery, BG Klinik, University of Tübingen, Tübingen, Germany; 2 Center for Musculoskeletal Surgery, Charité–University Medical Center Berlin, Berlin, Germany

**Keywords:** Hip impingement, Derotation, Torsional alignment, Long-leg axis, Anterior knee pain

## Abstract

**Introduction:**

Despite the fact that osteotomies around the knee represent well-established treatment options for the redistribution of loads and forces within and around the knee joint, unforeseen effects of these osteotomies on the remaining planes and adjacent joints are still to be defined. It was, therefore, the aim of this study to determine the influence of a distal femoral rotation osteotomy on the coronal limb alignment and on the ischiofemoral space of the hip joint.

**Materials and methods:**

Long-leg standing radiographs and CT-based torsional measurements of 27 patients undergoing supracondylar rotational osteotomies of the femur between 2012 and 2019 were obtained and utilized for the purpose of this study. Postoperative radiographs were obtained after union at the osteotomy site. The hip–knee–ankle angle (HKA), the mechanical lateral distal femur angle (mLDFA), and the ischiofemoral space were measured. Comparison between means was performed using the Wilcoxon–Mann–Whitney test.

**Results:**

Twenty-seven patients underwent isolated supracondylar external rotation osteotomy to reduce the overall antetorsion of the femur. The osteotomy resulted in a 2.4° ± 1.4° mean increase in HKA and 2.4 mm ± 1.7 mm increase in the ischiofemoral space (*p* < 0.001).

**Conclusion:**

Supracondylar external rotation osteotomy of the femur leads to valgisation of the coronal limb alignment and increases the ischiofemoral space. This is resultant to the reorientation of the femoral antecurvature and the femoral neck. When planning a rotational osteotomy of the lower limb, this should be appreciated and may also aid in the decision regarding osteotomy site.

## Introduction

Osteotomies around the knee represent powerful modalities for the treatment of bony deformities and degenerative joint disease. The intended effects of these osteotomies act on joints by redistributing loads and force vectors. Rotational osteotomies of the femur influence the overall femoral antetorsion and demonstrate a direct influence on both the knee and hip joints. The vectors of the quadriceps muscle are ultimately altered by a rotational osteotomy of the femur, subsequently influencing lateral force vectors acting on the patella. Furthermore, the orientation of the femoral neck in space is also influenced by femoral anteversion. Clear evidence linking torsional abnormalities of the femur to hip disease is present [3, 4, 10, 15].

It is, therefore, important to consider all possible effects of an osteotomy during surgical planning and expand planning beyond the plane of interest. This would reduce the likelihood of creating an unwanted conflict on a different level.

This study will be dealing with the influence of a distal supracondylar rotation osteotomy around the knee on both the coronal limb alignment and the ischiofemoral space of the hip. Given that the orientation of the curvature of the femur is likely to change during a rotation osteotomy, the question of whether the curvature may influence the coronal alignment is valid. Furthermore, the ischiofemoral space has been described as a conflict between the femur and the ischium and is gaining recognition as a cause of hip pain. High femoral antetorsion was shown to be associated with a reduced ischiofemoral space, due to which proximal torsional correction osteotomies have been proposed as efficient treatment options [4, 5, 9].

The aim of this study was to retrospectively determine the influence of supracondylar rotation osteotomies of the femur on the long-leg axis in the frontal plane and on the ischiofemoral space of the hip. We hypothesized that supracondylar external rotation osteotomy of the femur leads to valgisation of the long-leg axis and increase in the ischiofemoral space of the hip.

## Materials and methods

### Patients

**Fig. 1:**
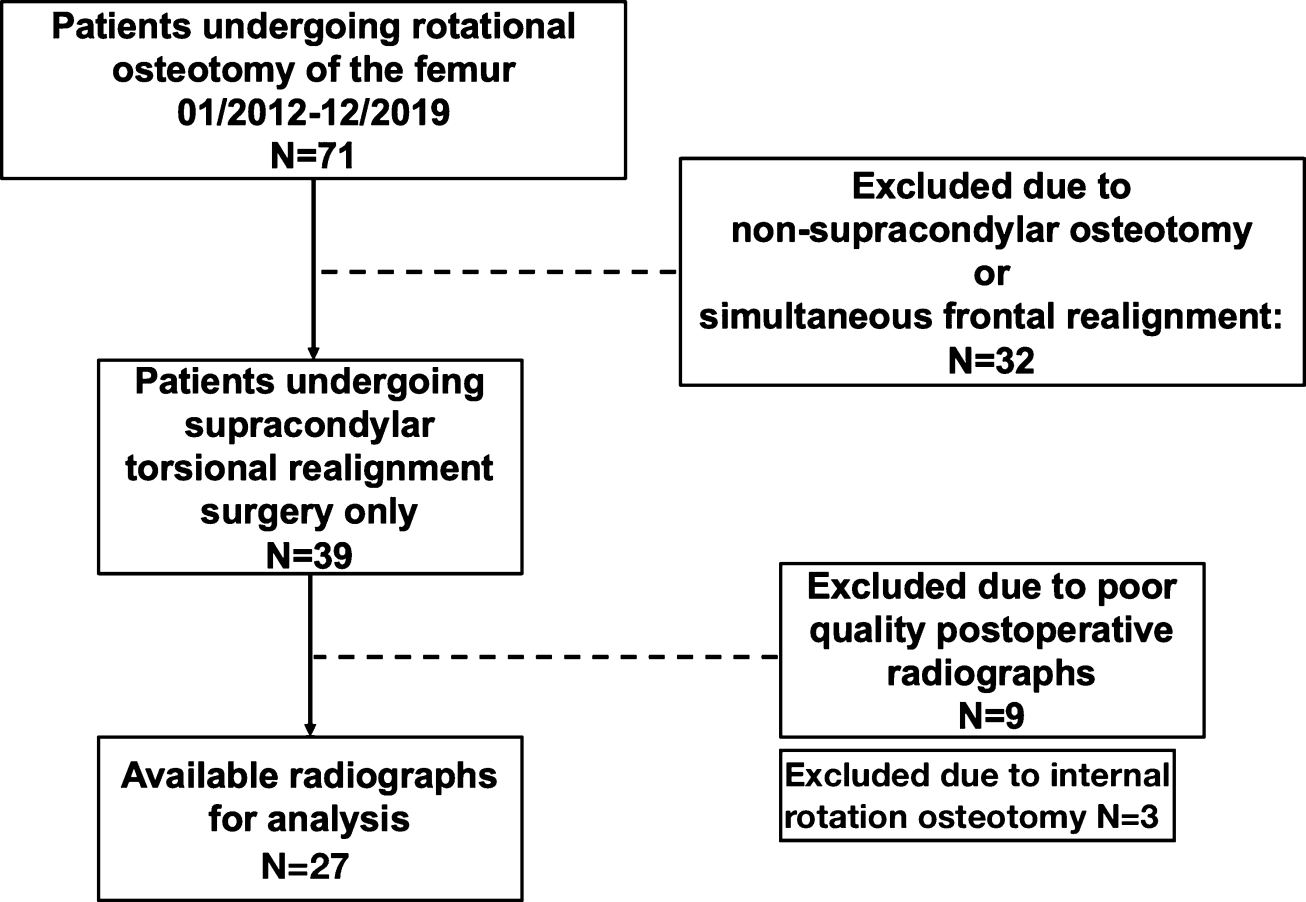
Flowchart demonstrating inclusion

Patients undergoing rotational osteotomy of the femur were considered eligible for inclusion in the study, provided that sufficient pre- and postoperative radiographs were available. Indication for surgery was patella maltracking with retropatellar pain in patients with femoral antetorsion (= femoral internal rotation) of more than 30°. Patients were excluded if a correction in a plane other than the axial plane was performed. No magnification device was present on the postoperative radiograph. Exclusion was necessary, if X-ray quality was defined as inferior, or in the case of missing consent regarding the utility of clinical data. Considering the above criteria, 27 legs of 26 patients undergoing osteotomy were considered eligible for inclusion in the study (Fig. 1).

### Surgical procedure

All osteotomies were planned using a landmark based deformity analysis [12, 14]. A medial subvastus approach was established [6, 21]. Supracondylar osteotomy was performed and a TomoFix MDF plate (DePuy Synthes, Solothurn, Switzerland) was used for fixation [2].

### Radiographs

Long-leg weight-bearing radiographs were obtained in accordance to Paley with a 1.3 m cassette (Global Imaging Baltimore, MD) [12]. Long-leg antero-posterior standing radiographs were obtained with the patient standing in a bipedal stance in front of the long film cassette. The radiography tube was positioned in a distance of 305 cm. The selected film cassette was of sufficient length to include the hips, knees, and ankles. The magnification with this setup was approximately 5%. A calibration device (250-mm steel ball) was used to calibrate the radiographs. The X-ray beam was centered on the level of the knee joints.

Radiologic technical assistants were instructed to position both legs with the patella centered between the femoral condyles. It was of ultimate importance to ensure a standardized radiography. Femoral torsion was measured using axial CT slides. As multiple methods for measuring femoral torsion exist [8, 13], instead of using the simpler method by Jarrett [7], we measured the femoral torsion according to Waidelich [22] because of its high reliability and especially because norm values are available for this method [17, 22].

All radiographic images were obtained prior to surgery for planning of the deformity correction and were repeated postoperatively after union at the osteotomy site and recovery of limp-free full weight bearing.

### Radiographic parameters

Radiographic parameters were determined by the first and last author with an accuracy of 0.1 mm using mediCAD® (Hectec, Landshut, Germany). Both orthopedic surgeons independently determined the radiographic parameters and repeated the assessments after 12 weeks. For the second phase, the order of the images was randomized to eliminate any bias from the first reading. The following parameters were assessed in accordance to Paley [12]:

Mechanical medial proximal tibial angle (mMPTA)

Mechanical lateral distal femoral angle (mLDFA)

Mechanical lateral proximal femoral angle (mLPFA)

Anatomic mechanical angle of the femur (AMA)

Femoral torsion

**Fig. 2:**
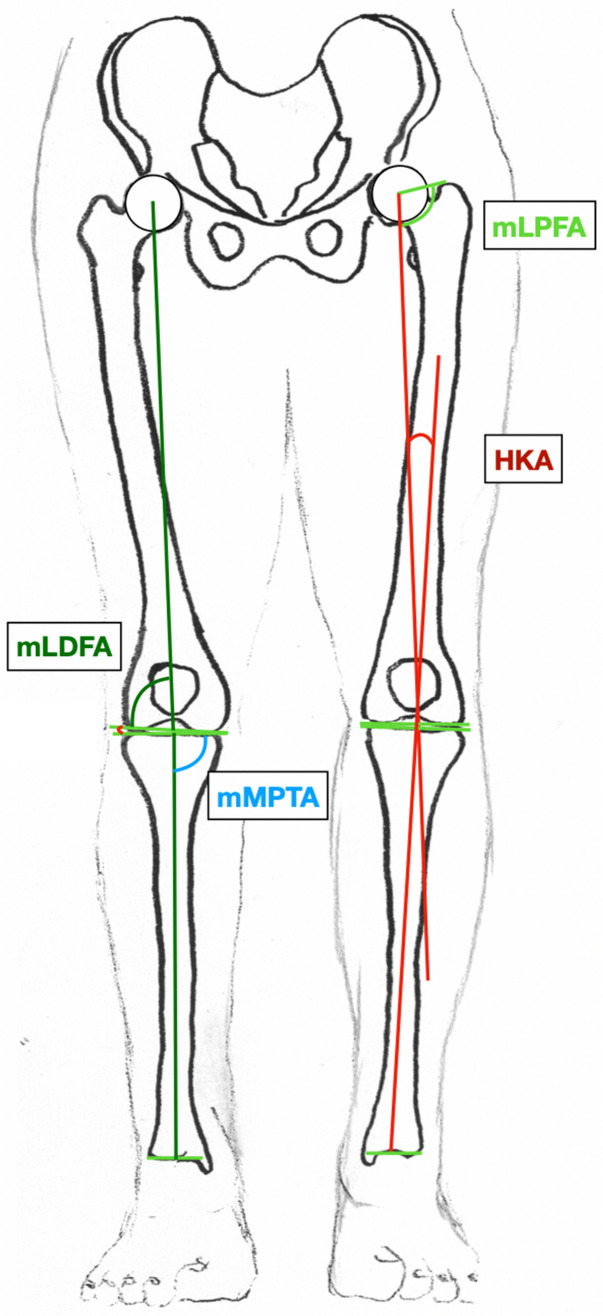
The radiographic parameters measured on a long-leg standing X-ray with the knees pointing forward. *HKA* hip–knee–ankle angle, *mLDFA* mechanical lateral distal femoral angle, *mLPFA* mechanical lateral proximal femoral angle, *mMPTA* mechanical medial proximal tibial angle

Hip–knee–ankle angle (HKA): refers to the angle between mechanic axes of the femur and the tibia (Fig. 2).

Moreover, the following radiographic measure was defined for the specific purpose of this study to address the primary research question:

#### Frontal ischiofemoral space

**Fig. 3:**
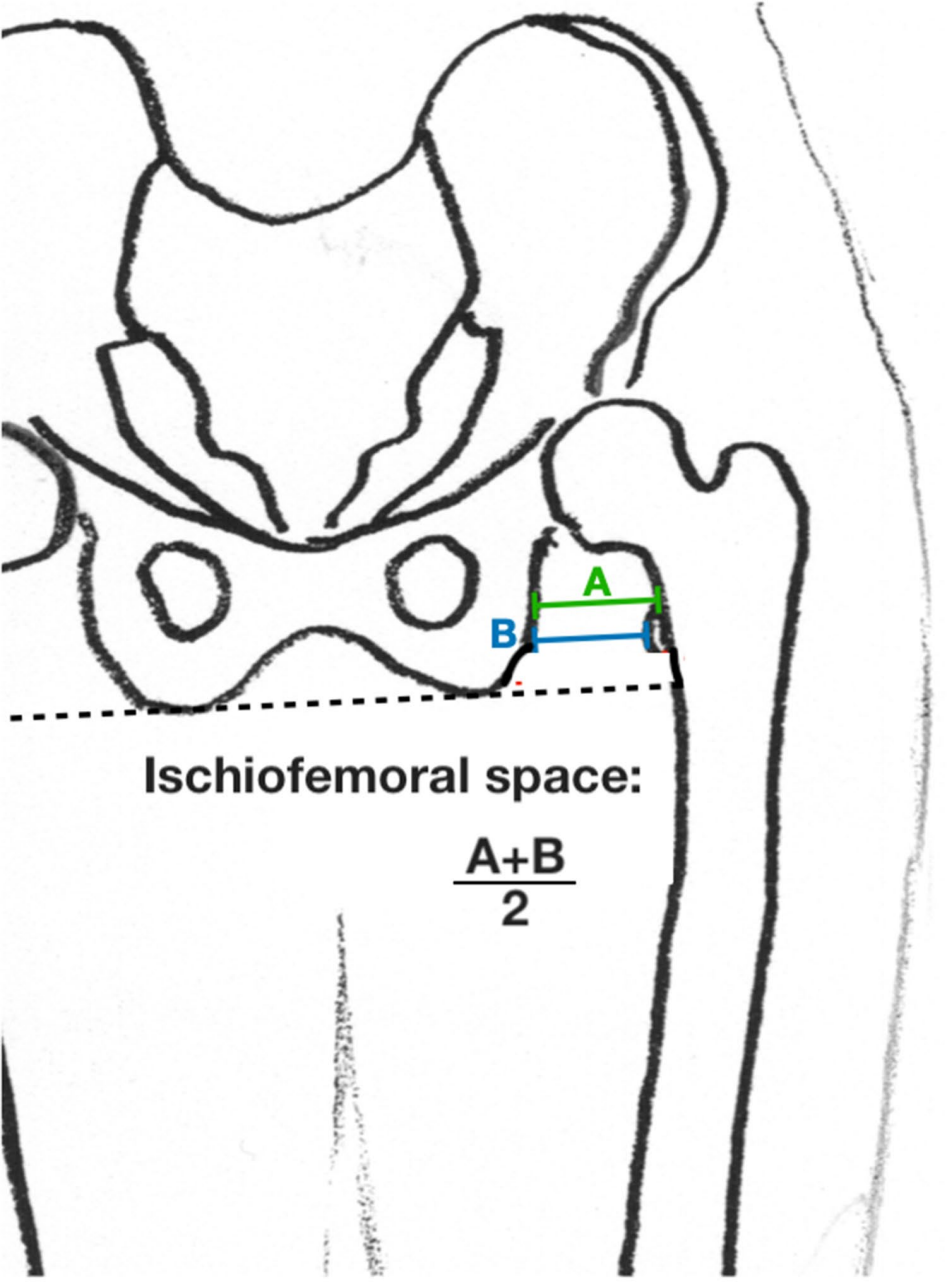
Frontal ischiofemoral space measurement on a long-leg standing X-ray with knees pointing forward

Which was defined as the mean of two distances measured between the femur and the ischium at the level of the lesser trochanter and in line with the pelvic orientation. The first distance runs between the lateral cortex of the ischium and the most superior portion of the lesser trochanter (A), the second was parallel to the first between the ischium and the most medial point of the lesser trochanter (B) (Fig. 3).

### Statistical analysis

Continuous variables were presented as mean ± standard deviation or range. Comparison between means was performed using the Wilcoxon test, because number of patients and data are limited and the test is non-parametric. A *p* value of < 0.05 was considered statistically significant. SPSS version 24 (IBM, Armonk, NY, USA) was used. A post hoc analysis was performed to ensure sufficient power to address the primary research question. Given the sample size of 27 patients, an effect size of 1.7 and an alpha error of 0.05, the power of the study was calculated to be 95%. The statistical analysis was supported by a statistician.

## Results

### Cohort demographics

The examined cohort of patients included 27 legs of 26 patients undergoing supracondylar external rotation osteotomy of the femur due to torsional malalignment of the femur and corresponding symptoms. The mean age was 32 (19–40) years. There were 21 female and 5 male patients.

### The effect of supracondylar external rotation osteotomy on bony alignment of the leg

Surgery lead to significant changes regarding femoral torsion, ischiofemoral distance, HKA, and mLDFA (Table 1). Other measures did not change significantly: AMA (6.60 ± 0.87 preop, 6.79 ± 0.45 postop, mLPFA (90.32 ± 2.49 preop, 90.23 ± 2.36 postop), mMPTA (87.31 ± 2.01 preop, 87.43 ± 2.60 postop).

**Table 1:** Radiographic measures in patients undergoing supracondylar external rotation osteotomy of the femur

Radiographic measure	Preoperative	Postoperative	∆	*p* value
Femoral torsion [°]	31.27 ± 4.00	17.43 ± 5.06	13.84°	< 0.001
Ischiofemoral distance [mm]	18.31 ± 4.67	20.67 ± 4.94	2.36 mm	< 0.001
Hip–knee–ankle angle [°]	– 2.40 ± 1.06	0.00 ± 1.26	2.40°	< 0.001
mLDFA [°]	89.30 ± 1.80	86.97 ± 2.21	– 2.33°	< 0.001
mMPTA [°]	87.31 ± 2.01	87.43 ± 2.60	0.12°	n. s

*mLDFA* mechanical lateral distal femoral angle, *mMPTA* mechanical medial proximal tibial angle (neg. = var.; pos. = valg.)

**Fig. 4:**
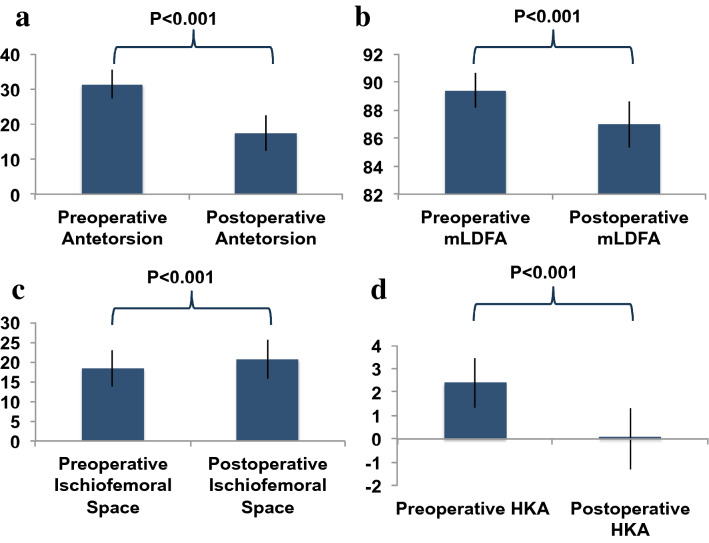
Supracondylar external rotation osteotomy of the femur: radiographic measures pre- and postoperatively. **a** Femoral antetorsion. **b** Ischiofemoral distance. **c** mLDFA. **d** HKA. *HKA*hip–knee–ankle angle, *mLDFA* mechanical lateral distal femoral angle

**Fig. 5:**
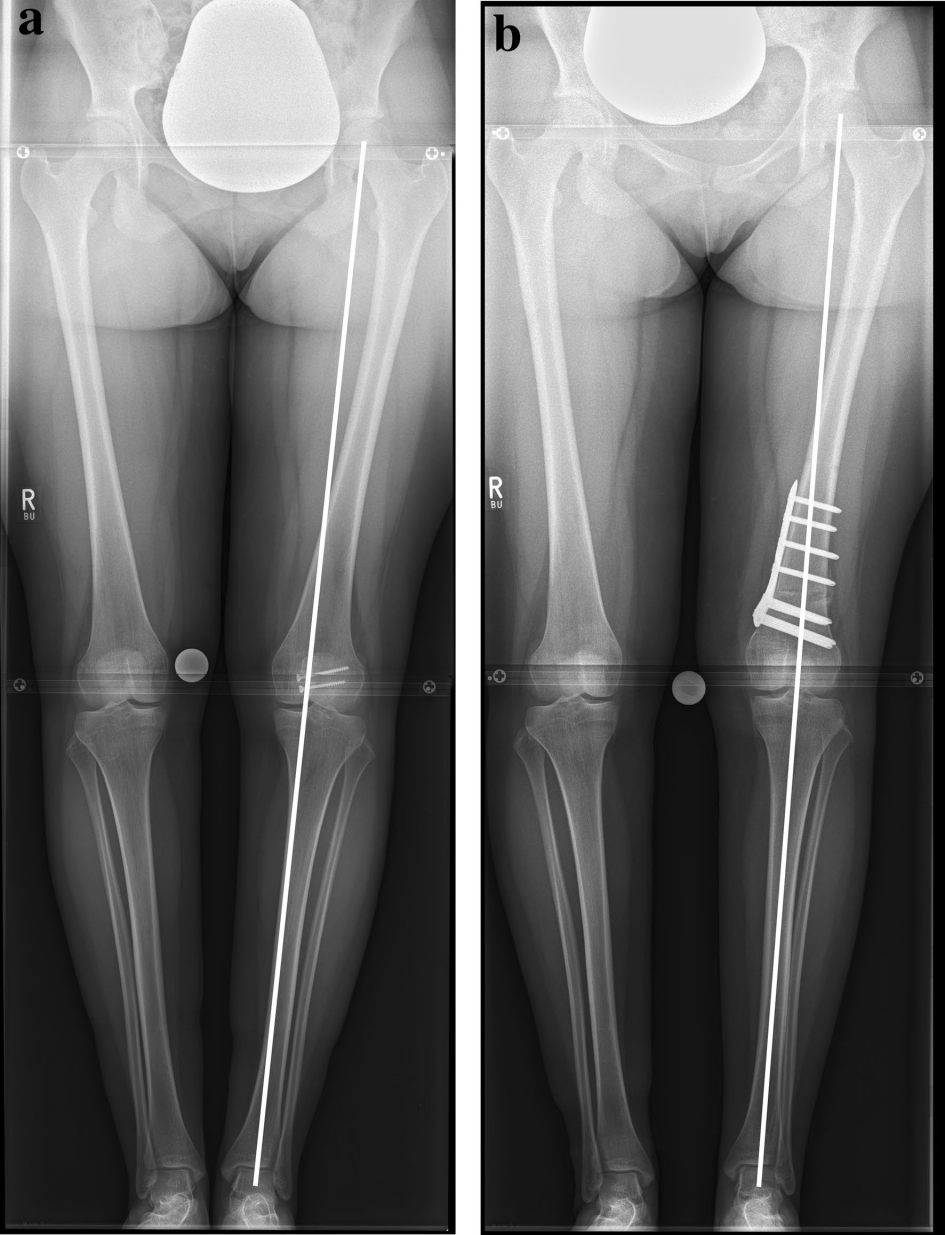
**a** Preoperative and b postoperative standing radiographs of a 20-year-old female patient. Isolated supracondylar external rotation osteotomy of the lift femur was performed with reduction of the femoral antetorsion from 30.0° to 15.8°. By this, the mLDFA decreased from 92.9° to 91.5° and the ischiofemoral space increased from 12 to 16 mm. Two screws were removed from the patella after an ipsilateral patella fracture in the past

Supracondylar external rotation osteotomy led to the following three measured changes, which are illustrated in Figs. 4a–d and 5a, b: (1) reduction of femoral antetorsion (*p* < 0.001), (2) increase of ischiofemoral distance (*p* < 0.001), and (3) valgisation of the long-leg axis (*p* < 0.001) as demonstrated by the HKA angle and the mLDFA (Table 1).

The intraobserver agreement was 89% for the first observer and 93% for the second observer. In addition, the interobserver agreement was high with a kappa-value of 77% for the first round of observations and 80% for the second round.

## Discussion

**Fig. 6:**
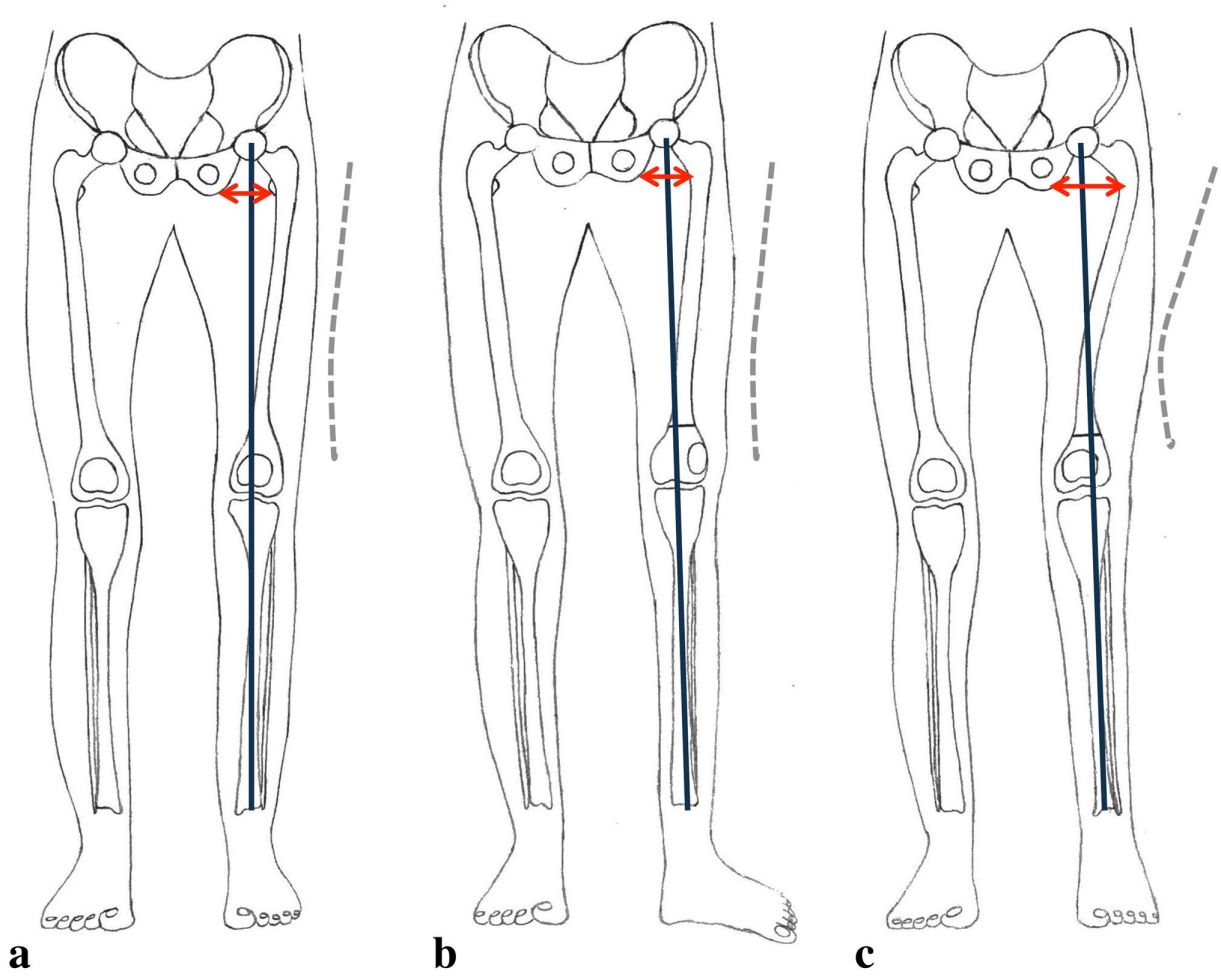
Three-dimensional changes of the lower extremity due to supracondylar external rotation osteotomy of the femur **a** pre-osteotomy, **b** post-osteotomy with hip joint in original rotation and foot pointed outwards, **c** post-osteotomy with hip in new position and foot directed forward: (1) decrease in antetorsion, (2) increase in ischiofemoral distance, and (3) valgisation of the long-leg axis. Black line mechanical axis weight-bearing line, dashed line femoral antecurvature orientation. red arrows line ischiofemoral distance

The most important findings of this study show that a supracondylar external rotation osteotomy increases not only the ischiofemoral space between the ischial tuberosity and the lesser trochanter, but also leads to a valgisation of the leg axis in the frontal plane. This is due to the resulting valgus alignment of the femur in the frontal plane. Figure 6 illustrates the effects of an external rotation osteotomy on the limb alignment and on the ischiofemoral space.

This study was based on the theory that a change in orientation of the femoral antecurvature could alter the axis of the limb. Given that a long-leg standing X-ray is performed with the knees pointing forward, it can be deduced that the X-ray represents a natural illustration of a standing position of the lower extremity. Therefore, one must understand that the hip and femur rotate internally in relation to the femoral condyles and the convex side of the femoral antecurvature rotates from anterior to medial, resulting in a reduction of the mLDFA in an external rotation osteotomy of the distal femur. A bony valgus alignment of the femur is, therefore, created. The results of this study confirm the fact, and therefore, the null hypothesis is rejected. In practice, supracondylar external rotational osteotomy was performed to reduce femoral antetorsion. On the operating table, external rotation of the distal femur fragment at first seemingly leads to a varisation of the leg on a two-plane X-ray, especially if the knee joint is slightly flexed. After osteotomy, as the patient walks with the foot directed straight forward, the patient rotates the hip joint inwards. As the osteotomy was performed distal to the antecurvature of the femur, the whole antecurvature also is rotated inwards and, as a matter of fact, this leads to a valgisation of the long-leg axis.

The clinical findings of the study may be seen when considering the site of the osteotomy. If valgisation is to be prevented, it would be helpful to perform rotational correction proximal to the level of the femoral antecurvature. An intertrochanteric osteotomy would have the same influence on the orientation of the femoral neck axis (femoral torsion), without changing the mLDFA. For example, an external rotation osteotomy in a patient with a valgus deviation should be performed proximal to the femoral antecurvature not to increase the valgus, whereas the same torsional deformity correction of the femur in a patient with a varus deformity should be performed distal to the femoral antecurvature at the supracondylar region. This will decrease the varus by valgisation.

Meticulous preoperative planning of the rotational osteotomy paying attention to all important angles especially near the knee joint is important to avoid unintentional creation of unphysiological angles outside the normal range.

The findings of this study are supported by an experimental study published earlier using a computer model with simulated external rotation osteotomies at different locations along the femur. The authors found a valgisation effect in distal (supracondylar) osteotomies. This alteration of the long-leg axis in the frontal plane was pronounced in cases with exceptional high femoral antecurvature [11].

Today, in the era of subspecialization, it is common practice for the hip surgeon to correct maltorsion of the femur proximally [1, 3, 10, 15, 16, 19]. The specialized knee surgeon might tend to correct the same deformity distally, at the supracondylar site [18, 20]. But this might not be the best option for every individual case given the fact that patients presenting with patellofemoral pain commonly have a valgus deviation, which should not be aggravated by external rotation osteotomy at the distal end of the femur.

Therefore, not only in symptomatic knees, but also in hip patients, the adjacent joint should be examined and the long-leg axis should be analyzed as part of the routine preoperative workup.

The main limitation of the study could be seen in the fact that the ischiofemoral distance was measured in the frontal plane using long-leg weight-bearing radiographs. A three-dimensional determination of the ischiofemoral space would provide a more accurate depiction. This would require complex imaging in a functional standing position. Given that the aim of this study was to prove the concept, the authors agreed on the sufficiency of the design chosen in this study. Another limitation is the relatively small patient cohort.

## Conclusions

Supracondylar external rotation osteotomy of the femur leads to valgisation of the coronal limb alignment and increases the ischiofemoral space. This is resultant to the reorientation of the femoral antecurvature and the femoral neck. When planning a rotation osteotomy of the lower limb, this should be appreciated and may also aid in the decision regarding osteotomy site.

## Abbreviations

AMA: Anatomic mechanical angle of the femur
HKA: Hip–knee–ankle angle
mLDFA: Mechanical lateral distal femoral angle
mLPFA: Mechanical lateral proximal femoral angle
mMPTA: Mechanical medial proximal tibial angle
